# Inter-rater reliability of hand motor function assessment in Parkinson’s disease: Impact of clinician training

**DOI:** 10.1016/j.prdoa.2024.100278

**Published:** 2024-10-28

**Authors:** Lorna Kenny, Zahra Azizi, Kevin Moore, Megan Alcock, Sarah Heywood, Agnes Johnson, Keith McGrath, Mary J. Foley, Brian Sweeney, Sean O’Sullivan, John Barton, Salvatore Tedesco, Marco Sica, Colum Crowe, Suzanne Timmons

**Affiliations:** a Centre for Gerontology and Rehabilitation, School of Medicine, University College Cork, Cork T12 XH60, Ireland; b Mercy University Hospital, Cork T12 WE28, Ireland; c Cork Stroke Support Centre, Cork T12 AKA4, Ireland; d Neurology Department, Bon Secours Hospital, Cork T12 DV56, Ireland; e Tyndall National Institute, University College Cork, Lee Maltings, Prospect Row, Cork T12 AV22, Ireland

**Keywords:** Parkinson’s disease, Assessment, Inter-rater reliability, Variability, MDS-UPDRS

## Abstract

Medication adjustments in Parkinson’s disease (PD) are driven by patient subjective report and clinicians’ rating of motor feature severity (such as bradykinesia and tremor).

**Objective:**

As patients may be seen by different clinicians at different visits, this study aims to determine the inter-rater reliability of upper limb motor function assessment among clinicians treating people with PD (PwPD).

**Methods:**

PwPD performed six standardised hand movements from the Movement Disorder Society’s Unified Parkinson’s Disease Rating Scale (MDS-UPDRS), while two cameras simultaneously recorded. Eight clinicians independently rated tremor and bradykinesia severity using a visual analogue scale. We compared intraclass correlation coefficient (ICC) before and after a training/calibration session where high-variance participant videos were reviewed and MDS-UPDRS instructions discussed.

**Results:**

In the first round, poor agreement was observed for most hand movements, with best agreement for resting tremor (ICC 0.66 bilaterally; right hand 95 % CI 0.50–0.82; left hand: 0.50–0.81). Postural tremor (left hand) had poor agreement (ICC 0.14; 95 % CI 0.04–0.33), as did wrist pronation-supination (right hand ICC 0.34; 95 % CI 0.19–0.56). In post-training rating exercises, agreements improved, especially for the right hand. Best agreement was observed for hand open-close ratings in the left hand (ICC 0.82, 95 % CI 0.64–0.94) and resting tremor in the right hand (ICC 0.92, 95 % CI 0.83–0.98). Discrimination between right and left hand features by raters also improved, except in resting tremor (disimprovement) and wrist pronation-supination (no change).

**Conclusions:**

Clinicians vary in rating video-recorded PD upper limb motor features, especially bradykinesia, but this can be improved somewhat with training.

## Highlights

• Clinicians showed variability in assessing Parkinson’s Disease motor features, with an overall inter-rater agreement (ICC) of 0.51.
• Variability in clinician assessments was reduced after a calibration session, which improved the overall ICC to 0.70.
• Following calibration, clinicians improved in independently rating hands, aiding assessment and lateralisation of PD motor features.
• Variability in ratings between clinicians can be moderated to some extent through a training and calibration session.

## 1 Introduction

Parkinson’s disease (PD) is a neurodegenerative disorder that causes progressive decline in motor function and non-motor symptoms [1], affecting over 8.5 million people worldwide [2].

The Movement Disorder Society's Unified PD Rating Scale (MDS-UPDRS) is the gold-standard clinical assessment tool for PD, providing instructions for evaluating hand function (part III of the scale), where patients perform standardised movements, and clinicians assign severity scores based on the degree of motor function impairment [3]. Reliability has been demonstrated in previous studies (e.g. Goetz et al., 2008) [4], including external validation [5], nonetheless, the subjective nature of motor function rating by clinicians introduces potential variations in interpretation, even when strictly adhering to the guidelines [6]. Utilising the scale correctly depends on the skills of clinicians, where variations in ratings can stem from individual biases and differing assessments of specific motor functions. As patients undergo evaluations by different clinicians at different visits, these divergent scores can influence the evaluation of disease progression or treatment response [6].

Inter-rater reliability (IRR) refers to the reliability of data collected by multiple evaluators who assess the same individuals during a single instance [7]. It helps determine whether a measurement instrument yields accurate results that clinicians can rely on to make decisions. While offering an impartial evaluation of motor severity, the IRR of the MDS-UPDRS poses possible issues, especially in multicentre trials for PD and in long-term studies involving multiple raters assessing the same patient at different intervals [4]. It may not be adequately reliable for tracking a patient over time due to psychometric concerns such as; measurement of motor symptoms and impact in early PD, and misalignment in MDS-UPDRS-III items implies a lack of sensitivity in detecting variations and clinical change [8]. The MDS-UPDRS-III change scores display considerable variability due to error (within-subject reliability ranged from 0.13 to 0.62), highlighting an urgency for more dependable assessment [9].

Accurate assessment of people with PD (PwPD) is vital for providing optimal care support and treatment [10], monitoring disease progression [11], and advancing research [10].

A number of technologies have been used for supporting clinicians with the assessment of motor symptoms in PwPD. These technologies vary from very low-cost and unobtrusive systems to expensive technology requiring the set-up of specific labs [12]. Examples could include digitalised tools (such as smart pens) for handwriting assessment [13], smartphones (equipped with inertial sensors, Global Positioning Systems, audio, and camera) for real-world assessment in free-living settings [14], force plates [15], and pressure mats/insoles for evaluation of the quality of gait [16], cameras for monitoring patients’ movements remotely at home or in controlled lab settings [17], motion capture systems (such as Vicon, or Microsoft Kinect) [18], physiological sensors (such as electrocardiogram, electromyography, electroencephalogram) [19], often combined within wearable sensors (generally equipped with inertial sensors) for monitoring remotely body movements, gait, and symptoms [20].

Going forward, PD care is likely to involve wearable devices [21], due to their low-cost, portability, and unobtrusiveness, and achieving accurate calibration depends on precise input from expert clinicians [22]. An online program offers teaching videos of the MDS-UPDRS and has shown to be an asset in improving both inter-rater and longitudinal reliability [23]. However, the effectiveness of learning may be impacted as videos are often watched in isolation and do not provide direct instructions or support. Interactive and team-based learning environments likely offer a more comprehensive experience and may foster deeper understandings [24]. Having direction and opportunity to ask questions in real time may be helpful for understanding and putting complex assessments like the MDS-UPDRS into practice [25], [26]. To the best of the authors knowledge, no studies have investigated the potential improvement in IRR through a group calibration exercise.

This study aimed to determine the IRR of hand motor function assessment among clinicians who treat individuals with PD at specialised clinics and whether a group calibration exercise could enhance IRR. This research is part of a broader European-funded initiative called SENDoc (Smart sENsor Devices fOr rehabilitation and Connected health). (https://sendoc.interreg-npa.eu/). The primary objective was to assess the precision of wearable devices in measuring hand motor functions in PwPD, with the goal of developing a novel device that captures PD-related health information. Part of assessing devices is understanding the accuracy of examination by experts, alongside current practice, and to what degree this can be improved.

## 2 Methods

### 2.1 Participants

PwPD were recruited through branches of the Parkinson’s Ireland and a specialised PD clinic in Munster, Ireland. Those interested received an information sheet about the study and researchers' contact details. Prospective participants contacted the team, allowing for detailed explanation of the study and the opportunity to ask questions. Inclusion criteria included individuals aged 50 and above with a confirmed PD diagnosis. A criterion-based theoretical sampling strategy ensured representation across different age categories: 50–60, 61–70, 71–80, and 80+, and a mix of male and female participants. Additionally, two spouses of a PwPD were invited to mimic a scenario where some patients have no abnormal upper limb motor status. Data collection occurred at Tyndall National Institute and St Finbarr’s Hospital in Cork, Ireland. Participants provided informed consent and access to their personal data. The study received ethical approval from the Clinical Research Ethics Committee at University College Cork, number ECM 4 (a) 16/10/19.

### 2.2 Data collection

Demographic and clinical information collected included participant details such as age, gender, height, weight, the currently affected side (right, left, or both), and PD stage determined by the Hoehn and Yahr. The number of years since diagnosis and the participant's current subjective ON/OFF state were recorded.

In the assessment phase of the research, participants were seated in a desk chair in the study site. Researchers attached electromyography electrodes to each palm, and on the back of each forearm. Inertial measurement units were attached to the back of each hand. Following highly standardised instructions (Table 1 in the Supplementary File), participants executed six hand movements from the motor section of the MDS-UPDRS-III. Tasks included; resting tremor for 30 s, postural tremor for 15 s, and kinetic tremor exercises, five times per hand. Additionally, participants performed 30 rapid finger taps per hand, 15 hand opening and closing movements per hand, and 10 wrist pronation and supination movements per hand, all with arms extended in front of their body.

Fig. 1 in the Supplementary File shows a participant performing a hand movement assessment. Movements were recorded simultaneously from a frontal and 45-degree angle perspectives (to ensure one arm did not obstruct the other) using two cameras.

To ensure anonymity, facial blurring was applied to videos. The six-hand movements were then presented to the raters, as a short video clip, in random order to minimise potential bias.

### 2.3 Raters and severity scoring

Raters with clinical experience of PD were recruited through regional networks known to the research team, and allowed for representation of individuals with diverse experiences. Eight clinicians, consisting of two geriatricians (who ran a specialised PD clinic), two neurologists (one with a special interest in PD; one highly experienced in neurology), one PD specialist nurse, and three senior geriatric registrars (with at least 3 months’ experience at a PD clinic), were initially recruited for the first round of data collection.

Raters independently reviewed the videos in accordance with the guidelines outlined by the MDS-UPDRS, and provided individual assessments for the severity of tremor and bradykinesia in each participant. Severity was marked in pen on a visual analogue scale (VAS) to generate continuous rather than categorical data. Some parts of the MDS-UPDRS, like rigidity and tremor amplitude, have clear criteria, making it easier for raters to agree. However, items related to bradykinesia, where terms like “slight”, “mild”, and “moderate” can lead to different interpretations among raters, and subsequently is more prone to variability [27]. The VAS was presented as a 100 mm black horizontal line on paper, with “Normal” printed to the left and “Severe” printed to the right, with no internal markings or numbers. Raters were instructed to draw a vertical line on the VAS to indicate severity ratings for each specific hand movement, separately for each hand (right and left) and for each motor task. Researchers measured the distance from the left edge of the VAS line to the rater-marked vertical line of severity, with measurements recorded to the nearest millimetre. Ratings placed at the far left or right of the line were recorded as 0 or 100, respectively.

Following analysis of the initial ratings, clinicians convened in a 2-hour “calibration” session to explore variance between raters. As a group, four participant videos exhibiting high variance were reviewed and the MDS-UPDRS instructions were discussed. Subsequently, 3–5 weeks later, six clinicians independently rated a second set of videos featuring 10 new participants. Two clinicians (one neurologist and one senior geriatric registrar) were unable to complete due to work unforeseen commitments.

### 2.4 Statistical analysis

The primary evaluation metric was the intraclass correlation coefficient (ICC), a widely adopted measure for determining IRR, and is utilised in studies focusing on IRR and agreement analysis [28]. ICC was calculated for each movement in every rating round, as an estimation of the level of agreement among raters, and was calculated for each movement using the iccNA function in the “irrna” package (v0.2.3) in R (R Core Team (2022) https://www.R-project.org/) and 95 % confidence intervals were reported. A 2-way random effects model without interaction was utilised, and the results for absolute agreement [ICC(A,1)] were extracted. Koo and Li (2016) categorise ICC values as; below 0.50: poor reliability; 0.50 to 0.75: moderate reliability; 0.75–0.90: good reliability; and above 0.90: excellent reliability. Generally, for routine motor assessments (e.g., bradykinesia, tremor), an ICC above 0.75 is often deemed clinically acceptable for research or diagnostic purposes. For critical diagnostic decisions or clinical trials, a higher ICC of above 0.90 is preferred [29]. Lower ICC values may reflect not only poor agreement between raters but also experimental errors, rater biases, or lack of variability among subjects [29]. For PD assessments in real-world settings, high IRR between clinicians assessing the same motor tasks is essential for reliable monitoring of the disease, and improved clinical decision making [7]. Wilcoxon signed tests were used to compare continuous variables across two rounds. The level of significance was established as 95 % (p < 0.05).

## 3 Results

The first round of ratings included 18 PwPD and 2 healthy controls, ranging in age from 55 to 81, Hoehn and Yahr (H&Y) stage 2–4, with 61.1 % (11/18) at stage 2. The predominantly affected body side was left in nine participants (self-report), right in six and bilateral in two, while one was unsure. For fourteen participants, all assessments were performed in a self-reported clear ON state; in two participants, they felt clearly OFF throughout; the remainder (n = 2) did not experience clear ON/OFF periods in their opinion.

The second round of ratings included ten PwPD, ranging in age from 60 to 80, at H&Y stage 1–3, with the right side of the body more often affected (6:4). Table 2 contains participant summary statistics. No statistical difference existed between the two groups in any of the following: age (Wilcoxon signed rank test, W = 96.5, P = 0.89), H&Y stage (W = 104.5, P = 0.45), and years since diagnosis (W = 102, P = 0.58).

**Table 2:** Participant demographics.

	**1st Round** **N = 20**	**2nd Round** **N = 10**
**Gender**		
Male	12	4
Female	8	6
**Age**		
Median	72	71
Range	55–81	60–80
**Weight (kg)**		
Median	73	71.5
Range	57–102	57–102
**PD specific features**	**N = 18**	**N = 10**
**Subjective ON/OFF State**		
ON	14	8
OFF	2	2
Does not experience/Not aware	2	0
**Self-reported affected side**		
Left	9	4
Right	6	6
Bilateral	2	0
Participant unsure	1	0
**Hoehn and Yahr Stage**		
1	0	2
2	11	5
3	5	3
4	2	0
**Years since diagnosis**		
Median	4	4.5
Range	1–14	1–14

**Fig. 2:**
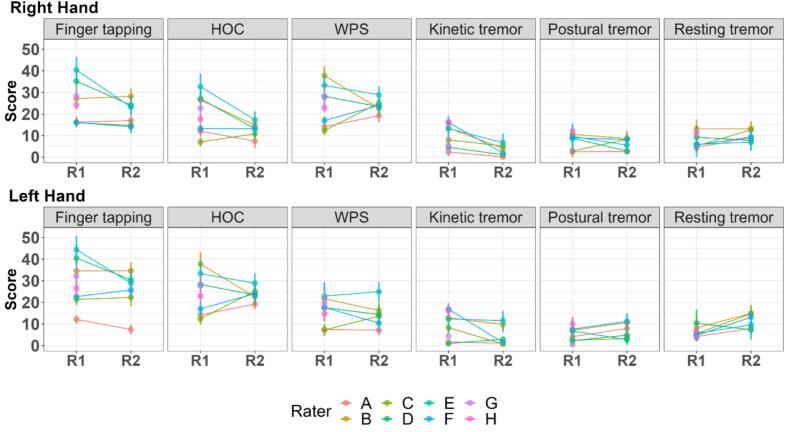
Mean severity score (arbitrary units), with standard error, from each rater for each motor task in round 1 (R1) and round 2 (R2), presented as data for right hands (above) and for left hands (below).

In round 1 (R1; n = 20 participants; n = 8 raters), mean severity scores (arbitrary units) from individual raters for assessments of bradykinesia-finger tapping, Hand Open-Close (HOC), and Wrist Pronation-Supination (WPS)-were higher than ratings for tremors, in both hands (right- and left-hand data presented separately; Fig. 2 in text and Table 3 in Supplementary File). In round 2 (R2; n = 10 participants; n = 6 raters), this difference is still present but less marked.

### 3.1 Agreement between raters

**Fig. 3:**
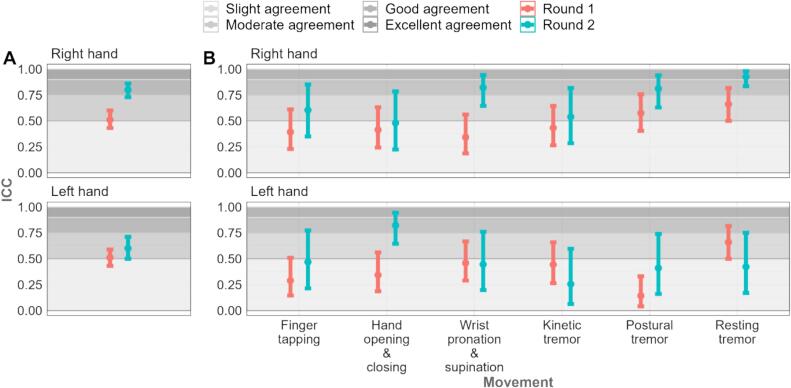
ICCs between raters for round 1 and round 2 for (A) all data (B) each movement. In both figures, data for the right hand is presented above, and the left hand below.

In round 1, there was moderate-to-poor agreement in ratings (ICC) among clinicians across the 240 discrete hand movement videos (overall ICC 0.51), with best agreement for resting tremors (right hand: ICC 0.66; 95 % CI 0.50–0.82; left hand: ICC 0.66; CI 0.50–0.81). Postural tremor in the left hand (ICC 0.14; 95 % CI 0.04–0.33) and WPS in the right hand (ICC 0.34; 95 % CI 0.19–0.56) had the least agreement (Fig. 3 in text and Table 4 in Supplementary File).

For post-training rating (round 2; n = 6 clinicians; 10 PwPD; 120 discrete hand movements) agreements improved generally (overall ICC 0.70), especially in the right hand (noting confidence intervals are wider due to smaller rater numbers; Fig. 3 in text; Table 4 in Supplementary Files). Best agreement was for HOC in the left hand (ICC 0.82; 0.64–0.94) and resting tremor in the right hand (ICC 0.92; 0.83–0.98). Least agreement was for kinetic tremor in the left hand (ICC 0.25; 0.06–0.60) and HOC in the right hand (ICC 0.48; 0.22–0.78).

### 3.2 Degree of relatedness in the ratings of hands

**Fig. 4:**
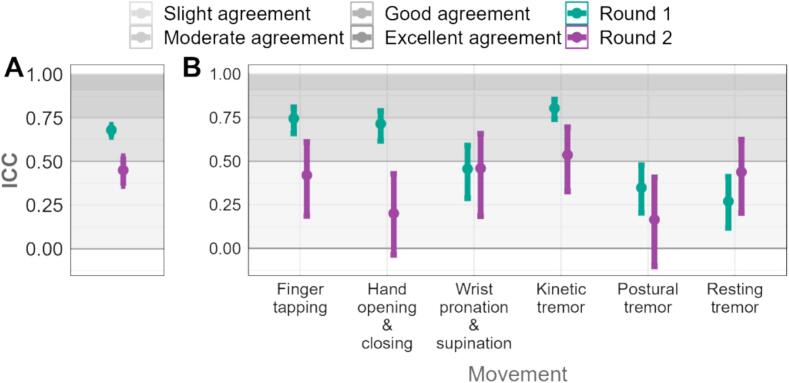
Relatedness of the severity scoring between two hands: (A) for all the data, (B) for each movement. A higher ICC indicates a greater relatedness between scores for the left and right hands in a single participant.

In round 1, there was a high degree of relatedness in the ratings of both hands for kinetic tremor severity (ICC 0.80; 95 % CI 0.74–0.85), and a moderate degree for finger tapping (ICC 0.74; 95 % CI 0.66–0.81) and HOC (ICC 0.71; 0.61–0.79). Therefore, raters appeared to judge both hands similarly (even though PD is by definition asymmetrical), and 15/18 participants identified a “worst” side. In round 2, this decreased significantly for three assessments. This may reflect participant characteristics (i.e., may have been more obvious asymmetry as not all identified a “worst” side) but suggests more independent rating by clinicians of each hand (Fig. 4in text and Table 5 in Supplementary File).

## 4 Discussion

This study shows substantial variability in experienced clinicians' assessments of PD motor features while examining video-recorded hand movements, where overall agreement (all movements combined) between raters was 0.51 (sub-item’s ICC ranged from 0.14 to 0.66). However, variability can be mitigated to an extent through a training and calibration session, resulting in overall ICC of 0.70, with a range from 0.25 to 0.92.

Although earlier studies show the ICC coefficients among MDS-UPDRS movements were high (ICC 0.90; [30])), IRR exhibited a variation among different studies. Richards et al. (1994) tested the IRR of the UPDRS (precursor to the MDS-UPDRS) [25] and reported a high ICC for resting tremor (0.84) but interestingly, the movement with best reliability was wrist pronation and supination which contrasts with our study where this movement displayed one of the least reliable assessments. Overall, their ICCs were higher than ours for similar movements. Meanwhile, there was only moderate agreement for finger tapping rating between neurologists (ICC 0.53; [31]). Also, Morinan et al. (2023) tested bradykinesia from the MDS-UPDRS encompassing various motor movement ratings [32].

Poorer agreement in our study may be attributed to several factors. Firstly, and importantly, previous studies used the ordinal 0–4 scale of the MDS-UPDRS, whereas we used continuous data on a VAS scale, which is arguably a more “pure” data measure where differences between raters are more apparent. Secondly, previous findings demonstrated a greater variation in rater scores when a limited number of movements were assessed in isolation, rather than alongside other clinical assessments [33], such as our study. Thirdly, in some of these studies [25], [34], few raters are used (4 and 3, respectively), compared to eight in ours, although other studies had larger panels of raters (e.g. 15 in Morinan et al., 2023 [32]).

Following group calibration clinicians reported a more independent rating of each hand. Most PD is asymmetric (i.e., idiopathic PD) and even when bilateral, the initially/worst affected side remains most affected [35]. This lateralisation is diagnostically important in suggesting PD aetiology (i.e. typical; versus atypical or more likely drug-induced), and right side predominant motor features have a more rapid progression [36]. Meanwhile, patients with their dominant side more affected have significantly poorer postural control [37]. Thus, ability to lateralise motor features is prognostically important, and so ability to improve left–right motor feature discrimination through training is a bonus. Several studies using ordinal scales have reported variability in motor symptom assessments among clinicians. For instance, Ren et al. (2021) and Goetz et al. (2008) emphasize the challenges of achieving consistent ratings with traditional ordinal scales [4], [38]. Our findings align with this research, extending it by demonstrating that VAS, which provide continuous and granular data, demonstrate significant inter-rater variability. VAS are commonly used clinically for assessing pain [39] and quality of life (e.g. EuroQol’s EQ5D). Literature indicates that both ordinal and VAS outcomes are susceptible to subjective interpretation in assessing individuals with PD, across domain such as pain [40], global severity [41], and quality of life [42]. An advantage of VAS in research, and also clinically, is its ability to detect subtle symptom severity graduations and to avoid language that might cause rater bias (such as “severe”). While two assessors may both choose “moderate” for severity of a motor task (and hence be judged to rate the same), a VAS can reveal differences in rating if one scores 50 for example and another 70. In our study, following training, IRR significantly improved for movements such as hand open-close and resting tremor, mirroring findings by Goetz et al. (2008) that training enhances reliability for ordinal scales. Notably, our results revealed non-uniform effects in resting tremor ratings post-training, specifically, one hand may exhibit an agreement, while the other may show a decrease, diverging from previous studies that typically report uniform improvements [4]. Ultimately, while VAS offers continuous data sensitive to subtle changes, which is useful for research, ordinal scales may be easier to rate and compare in clinical practice.

The MDS-UPDRS is recognised globally in routine clinical practice and is the most commonly accepted reference standard for assessing PwPD, providing clear categorical data that is easy to analyse, familiar, and consistent. Despite already stated limitations in categorical scales, it is still the most preferred approach to assessing PD for many. Given this already established status, prioritising training in the proper use is especially justified to ensure clinicians use consistently [43]. Our findings support this, as after a group calibration exercise, generally the ICCs improved. The most difficult case to rate is the subject with least impairment and raters had the greatest difficulty with the mildest impairment, revealing that training is especially important in early PD [43]. Conversely, we found that the lower the mean scores for a movement, the higher the apparent ICC; for example, resting tremor. Reasons for this are not clear.

Enhancing IRR in PD assessments can support consistent identification of key symptoms like tremor, bradykinesia, and rigidity, leading to timely diagnoses, better interventions, and improved outcomes [10], [11], [26]. Reliable symptom assessment ensures accurate medication dose adjustments [44], and supports therapeutic decisions like deep-brain stimulation (DBS) candidacy, where a high IRR is crucial for assessing levodopa response, typically requiring a 33 % improvement in MDS-UPDRS scores [45]. Additionally, improving IRR enhances multidisciplinary care by aligning specialists on treatment needs. However, clinical assessments may miss the slight variations in movement that are indicative of early-stage PD, or overlook fluctuations in symptoms and real-life functioning. It is likely that objective, device-aided monitoring would be needed for such precision.

Technology and low-cost computer systems [12], [13], [14], [15], [16], [17], [18], [19], [20] offer an opportunity to improve IRR in PD care by reducing subjective variations. These systems can augment consistent assessments across clinical locations, and raters, minimising human error. They also offer feedback for clinician training and ensure reliable data collection, improving the consistency of care. While some of the technologies have shown promising results in terms of feasibility and accuracy, their selection by clinicians depends on the specifications required for specific clinical scenarios and end-users (e.g., remote monitoring, cost, patients’ digital skills, assessment frequency).

## 5 Limitations

The study included a limited number of participants; a larger sample would provide greater understanding of IRR. The distribution of severity among participants was not Gaussian (normal), and the majority had mild to moderate severity PD (people with severe disease found it more challenging to participate), which may limit applicability to more advanced stages. Older people, minorities, and those with comorbidities are often underrepresented in research, limiting the ability to generalise study results [46]. Expanding the findings to a more diverse population could provide deeper insights into motor assessment variability in PD, as cultural and demographic factors affect disease presentation and access to care. There was a gender difference between the rounds, highlighting a potential variability that should be considered. Raters were mostly from one region which might limit the variety of experiences, and although there was a reasonable mix of expertise/discipline (three trainees, four consultants, one nurse), raters were more representative of geriatric medicine than neurology.

Two raters withdrew for the second round due to unforeseen professional commitments, and the second round had less participants (i.e., 8 raters rated 20 participants, and then 6 rated 10 participants). To ensure reliability, we compared the ICC from the same six raters in round 1 to the ICC from these six raters in round 2. Overall ICC for the six raters in round 1 was 0.46 (95 % CI [0.39–0.53]) and 0.70 (95 % CI [0.64–0.77]) in round 2. The trend of improvement from round 1 to round 2 is illustrated in Fig. 5 and Table 6 in the Supplementary File, and confirms that reliability of the raters was consistent across both rounds, independent of the number of raters.

The calibration session effectively addressed variations, however, it is unclear if a single 2-hour session would ensure long-term improvement in how consistently raters assess. Four participant videos were reviewed, which may not fully represent the diversity of cases, and rating a video may not be the same as rating face-to-face in real-time.

## 6 Future works

Clinicians' agreement improved after a training session, and relatedness between hands decreased, indicating an ability to focus on each side separately. Future work could target different training modalities and timeframes to examine the possibility of maintaining agreement between experienced clinicians over time. Scaling up of clinician PD training across disciplines would require careful planning and collaboration. Evaluator training, experience, and commitment to improving assessment quality are key factors influencing inter-rater evaluation [47]. Any future implementation of a training intervention for PD assessment should asses the long-term retention of the training effects and the scalability across diverse healthcare settings (e.g. from specialised centres to primary care) and across clinician experience levels. As research shows that knowledge decay can occur without reinforcement strategies [48], follow-up refresher courses may be necessary for sustained effectiveness. There is also scope to further explore the role of technology to improve both PD assessment reliability and overall care quality, including its acceptability and feasibility in busy clinic settings.

## 7 Conclusion

This study demonstrates significant variability in ratings between experienced clinicians when assessing video-recorded hand movements. This can be mitigated to some extent with a training and calibration session. Although improvements were observed, even after training, variability in ratings persisted and suggests that assessment of motor features remains complex, emphasising the importance of improving consistency of clinical assessments in PD, including the role of technology-enhanced assessments.

## 8 Ethical compliance statement

Ethical approval was obtained from the Clinical Research Ethics Committee of the University College, Cork, ECM 4 (a) 16/10/19.

Informed written consent was obtained from participants for their involvement in the research and for access to their personal data. Participants were provided with information leaflets detailing the study objectives, had the opportunity to pose questions, and subsequently signed a consent form, with both participant and researcher retaining a copy.

## 9 Funding statement

This work was supported by the Interreg Northern Periphery and Artic Programme funded project SENDOC (Smart sENsor Devices fOr rehabilitation and Connected health).

## CRediT authorship contribution statement

**Lorna Kenny:** Writing – review & editing, Writing – original draft, Visualization, Validation, Methodology, Investigation, Formal analysis, Data curation. **Zahra Azizi:** Writing – review & editing, Writing – original draft, Visualization, Validation, Formal analysis, Data curation. **Kevin Moore:** Writing – review & editing, Methodology, Data curation. **Megan Alcock:** Writing – review & editing, Methodology, Investigation. **Sarah Heywood:** Writing – review & editing, Methodology, Investigation. **Agnes Johnson:** Writing – review & editing, Methodology, Investigation. **Keith McGrath:** Writing – review & editing, Methodology, Investigation. **Mary J. Foley:** Writing – review & editing, Methodology, Investigation. **Brian Sweeney:** Writing – review & editing, Methodology, Investigation. **Sean O’Sullivan:** Writing – review & editing, Methodology, Investigation. **John Barton:** Writing – review & editing, Software, Methodology, Funding acquisition. **Salvatore Tedesco:** Writing – review & editing, Software, Methodology. **Marco Sica:** Writing – review & editing, Methodology, Investigation. **Colum Crowe:** Writing – review & editing, Methodology, Investigation. **Suzanne Timmons:** Writing – review & editing, Writing – original draft, Supervision, Conceptualization.

## Declaration of competing interest

The authors declare that they have no known competing financial interests or personal relationships that could have appeared to influence the work reported in this paper.

## Appendix A Supplementary data

Supplementary Data 1 Participant preforming a hand movement assessment.

Supplementary Data 2 ICCs between raters for round 1 and round 2 for (A) all data (B) each movement. In these figures, data for the right hand is presented above and the left hand below, and we only considered the same six raters in both rounds.
