# Supplementary figures and images for: Inter-rater reliability of hand motor function assessment in Parkinson’s disease: Impact of clinician training
Source: Clin Park Relat Disord. 2024 Oct 28;11:100278. doi: 10.1016/j.prdoa.2024.100278 (PMC11566327; doi:10.1016/j.prdoa.2024.100278)

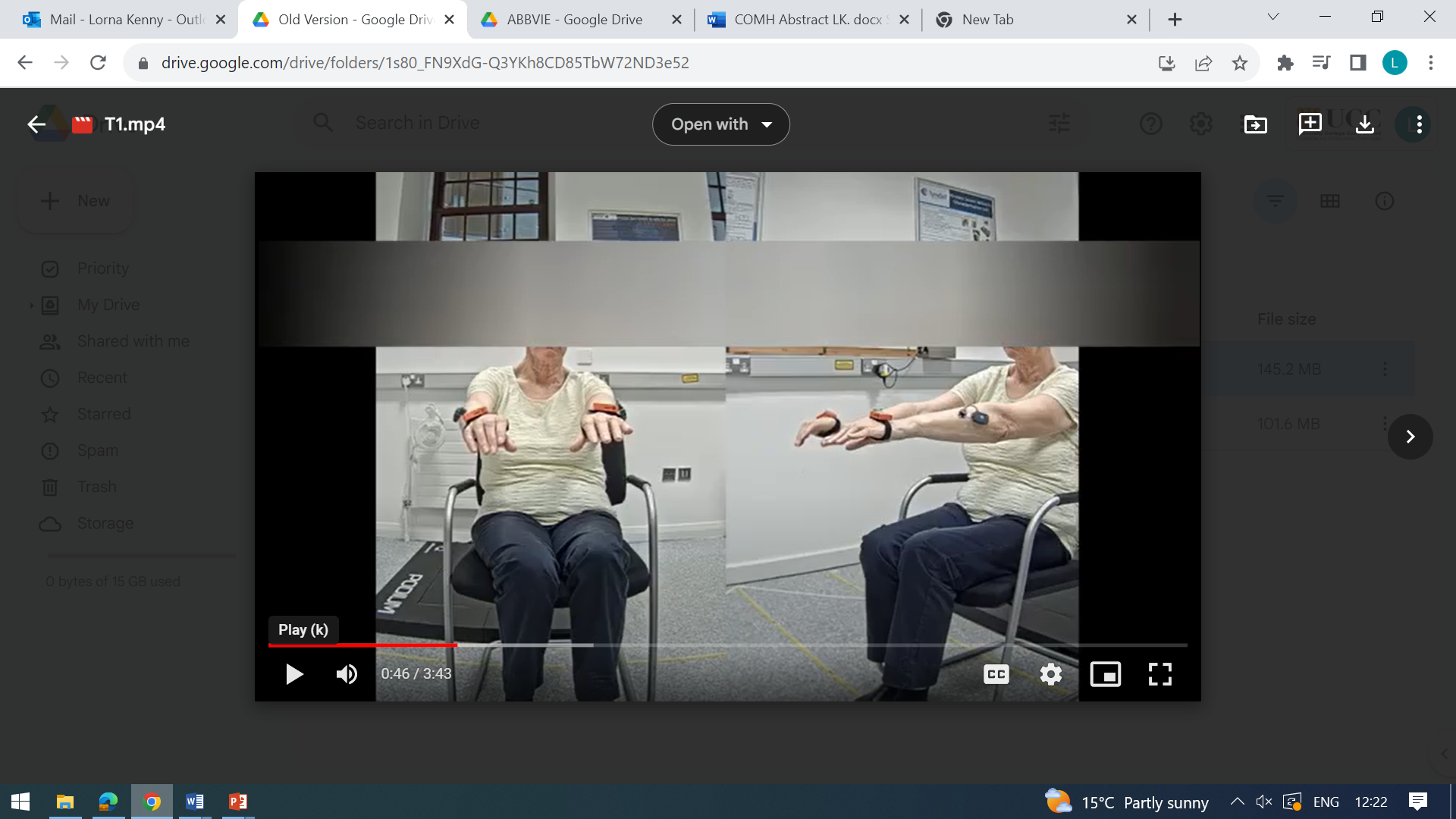

Supplement: Supplementary Data 1 — Participant preforming a hand movement assessment. [file mmc1.docx]

**
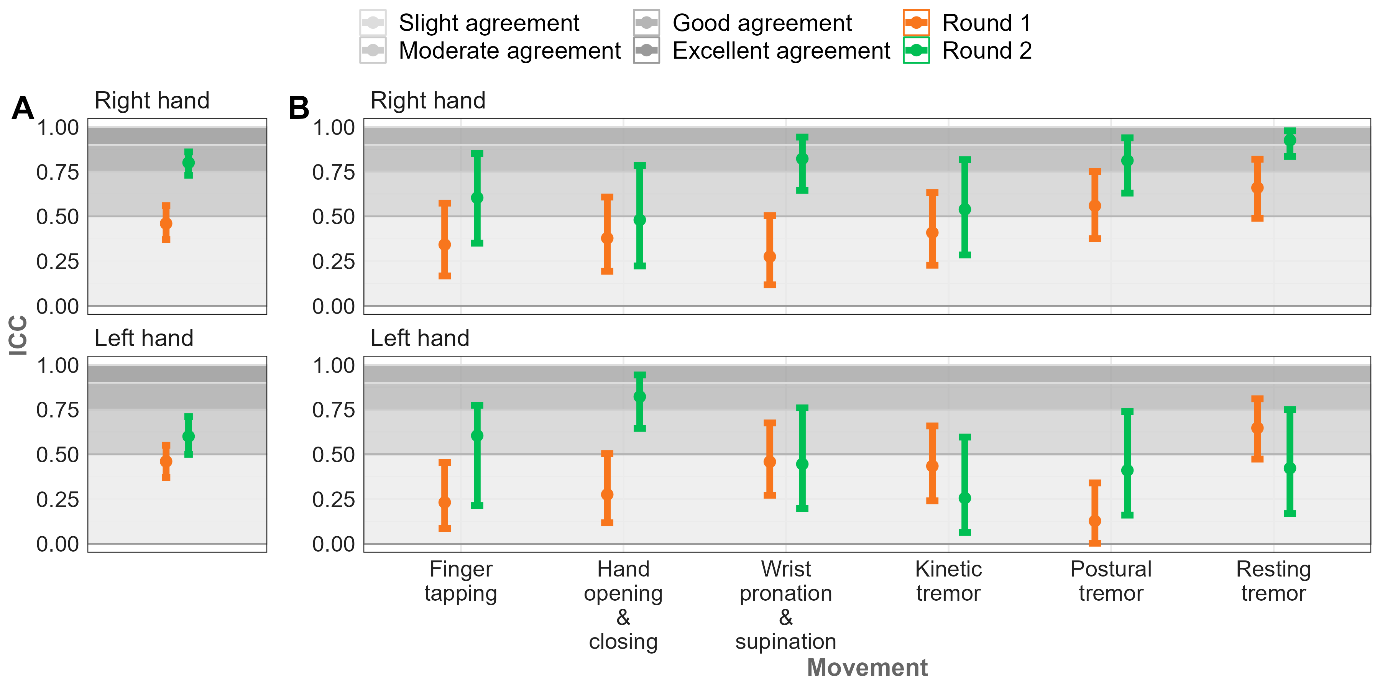
**

Supplement: Supplementary Data 2 — ICCs between raters for round 1 and round 2 for (A) all data (B) each movement. In these figures, data for the right hand is presented above and the left hand below, and we only considered the same six raters in both rounds. [file mmc2.docx]
