# Supplementary material for: Inter-rater reliability of hand motor function assessment in Parkinson’s disease: Impact of clinician training
Source: Clin Park Relat Disord. 2024 Oct 28;11:100278. doi: 10.1016/j.prdoa.2024.100278 (PMC11566327; doi:10.1016/j.prdoa.2024.100278)
Supplement: Supplementary Data 3 [file mmc3.docx]

| Name | Description | Repetitions |
| --- | --- | --- |
| Resting tremor | Participant sitting in chair, forearms resting on the arms of the chair, wrists hanging relaxed (not on their lap), and feet on floor. | Hold for 30 seconds |
| Postural tremor | Participant holding both arms out in front of their body at chest level, elbows straight, with palms facing down and fingers extended. | Hold for 15 seconds |
| Kinetic tremor | MO Tripod (approx. 80 cm-100cm tall) placed at arm’s length in front of the seated participants. Participants to extend their arm, touch the top of the tripod (green marker on top) with their index finger, and then touch their chest. Repeat with other arm. | 5 per hand |
| Finger tapping | With one arm extended in front of their body, participants tapped their index finger to their thumb as quickly and as wide as possible. Repeat with other arm. | 30 taps per hand |
| Hand opening and closing | With one arm extended in front of their body, elbows straight and palm facing the floor, participants made a fist and then stretched their hand flat, as fast and as fully as possible. Repeat with other arm. | 15 per hand |
| Wrist pronation and supination | With both arms extended in front of their body, elbows straight and palm facing the floor, participants turned their palms facing upwards and then downwards as fast and as fully as possible. | 10 per hand |

**Table 1:** Standardised hand movements for each participant
