# Supplementary material for: Inter-rater reliability of hand motor function assessment in Parkinson’s disease: Impact of clinician training
Source: Clin Park Relat Disord. 2024 Oct 28;11:100278. doi: 10.1016/j.prdoa.2024.100278 (PMC11566327; doi:10.1016/j.prdoa.2024.100278)
Supplement: Supplementary Data 4 [file mmc4.docx]

| **Movement** | **Round** | **Rating (± se)** |
| --- | --- | --- |
| Finger tapping | R1 | 27.36 (±6.38) |
|  | R2 | 22.83 (±5.47) |
| Hand opening and closing | R1 | 22.03 (±5.92) |
|  | R2 | 18.42 (±4.81) |
| Wrist pronation and supination | R1 | 20.17 (±5.68) |
|  | R2 | 19.01 (±5.31) |
| Kinetic tremor | R1 | 9.49 (±4.19) |
|  | R2 | 3.96 (±2.11) |
| Postural tremor | R1 | 6.21 (±3.12) |
|  | R2 | 6.38 (±4.37) |
| Resting tremor | R1 | 6.71 (±3.86) |
|  | R2 | 10.46 (±5.35) |

**Table 3:** Mean severity score, with standard error, from each rater for each motor task in round 1 (R1) and round 2 (R2)
