# Supplementary material for: Inter-rater reliability of hand motor function assessment in Parkinson’s disease: Impact of clinician training
Source: Clin Park Relat Disord. 2024 Oct 28;11:100278. doi: 10.1016/j.prdoa.2024.100278 (PMC11566327; doi:10.1016/j.prdoa.2024.100278)
Supplement: Supplementary Data 5 [file mmc5.docx]

| **Hand** | **Movement** | **Round** | **ICC** | **Lower CI** | **Upper CI** | ***p*** |
| --- | --- | --- | --- | --- | --- | --- |
| **Right** | Resting Tremor | round 1 | 0.661 | 0.501 | 0.816 | <0.001 |
|  |  | round 2 | 0.925 | 0.835 | 0.978 | <0.001 |
|  | Postural Tremor | round 1 | 0.575 | 0.403 | 0.758 | <0.001 |
|  |  | round 2 | 0.812 | 0.63 | 0.939 | <0.001 |
|  | Kinetic Tremor | round 1 | 0.432 | 0.263 | 0.645 | <0.001 |
|  |  | round 2 | 0.539 | 0.284 | 0.817 | <0.001 |
|  | Finger Tapping | round 1 | 0.393 | 0.228 | 0.611 | <0.001 |
|  |  | round 2 | 0.604 | 0.350 | 0.851 | <0.001 |
|  | Hand Opening & Closing | round 1 | 0.414 | 0.244 | 0.631 | <0.001 |
|  |  | round 2 | 0.48 | 0.223 | 0.784 | <0.001 |
|  | Wrist Pronation Supination | round 1 | 0.342 | 0.186 | 0.562 | <0.001 |
|  |  | round 2 | 0.822 | 0.645 | 0.943 | <0.001 |
| **Left** | Resting Tremor | round 1 | 0.659 | 0.5 | 0.815 | <0.001 |
|  |  | round 2 | 0.422 | 0.168 | 0.749 | <0.001 |
|  | Postural Tremor | round 1 | 0.142 | 0.04 | 0.142 | <0.001 |
|  |  | round 2 | 0.41 | 0.16 | 0.41 | <0.001 |
|  | Kinetic Tremor | round 1 | 0.443 | 0.264 | 0.658 | <0.001 |
|  |  | round 2 | 0.255 | 0.063 | 0.596 | <0.001 |
|  | Finger Tapping | round 1 | 0.289 | 0.144 | 0.508 | <0.001 |
|  |  | round 2 | 0.468 | 0.213 | 0.774 | <0.001 |
|  | Hand Opening & Closing | round 1 | 0.342 | 0.186 | 0.562 | <0.001 |
|  |  | round 2 | 0.822 | 0.645 | 0.943 | <0.001 |
|  | Wrist Pronation Supination | round 1 | 0.458 | 0.289 | 0.667 | <0.001 |
|  |  | round 2 | 0.445 | 0.197 | 0.759 | <0.001 |

**Table 4:** ICCs between raters for round 1 and round 2 for each movement, for the right hand and the left hand
