# Supplementary material for: Inter-rater reliability of hand motor function assessment in Parkinson’s disease: Impact of clinician training
Source: Clin Park Relat Disord. 2024 Oct 28;11:100278. doi: 10.1016/j.prdoa.2024.100278 (PMC11566327; doi:10.1016/j.prdoa.2024.100278)
Supplement: Supplementary Data 6 [file mmc6.docx]

| **Movement** | **Round** | **ICC** | **Lower CI** | **Upper CI** | ***p*** |
| --- | --- | --- | --- | --- | --- |
| Resting Tremor | round 1 | 0.27 | 0.12 | 0.41 | <0.001 |
|  | round 2 | 0.44 | 0.20 | 0.62 | <0.001 |
| Postural Tremor | round 1 | 0.35 | 0.20 | 0.48 | <0.001 |
|  | round 2 | 0.16 | -0.10 | 0.41 | 0.11 |
| Kinetic Tremor | round 1 | 0.80 | 0.74 | 0.85 | <0.001 |
|  | round 2 | 0.54 | 0.33 | 0.69 | <0.001 |
| Finger Tapping | round 1 | 0.74 | 0.66 | 0.81 | <0.001 |
|  | round 2 | 0.42 | 0.19 | 0.61 | <0.001 |
| Hand Opening & Closing | round 1 | 0.71 | 0.62 | 0.79 | <0.001 |
|  | round 2 | 0.20 | -0.04 | 0.43 | 0.04 |
| Wrist Pronation Supination | round 1 | 0.46 | 0.29 | 0.59 | <0.001 |
|  | round 2 | 0.46 | 0.18 | 0.66 | <0.001 |

**Table 5:** Relatedness between two hands
