# Supplementary material for: Inter-rater reliability of hand motor function assessment in Parkinson’s disease: Impact of clinician training
Source: Clin Park Relat Disord. 2024 Oct 28;11:100278. doi: 10.1016/j.prdoa.2024.100278 (PMC11566327; doi:10.1016/j.prdoa.2024.100278)
Supplement: Supplementary Data 7 [file mmc7.docx]

| **Hand** | **Movement** | **Round** | **ICC** | **Lower CI** | **Upper CI** | ***p*** |
| --- | --- | --- | --- | --- | --- | --- |
| **Right** | Resting Tremor | round 1 | 0.660 | 0.489 | 0.819 | <0.001 |
|  |  | round 2 | 0.925 | 0.835 | 0.978 | <0.001 |
|  | Postural Tremor | round 1 | 0.559 | 0.375 | 0.75 | <0.001 |
|  |  | round 2 | 0.812 | 0.63 | 0.939 | <0.001 |
|  | Kinetic Tremor | round 1 | 0.409 | 0.227 | 0.633 | <0.001 |
|  |  | round 2 | 0.539 | 0.284 | 0.817 | <0.001 |
|  | Finger Tapping | round 1 | 0.342 | 0.167 | 0.574 | <0.001 |
|  |  | round 2 | 0.604 | 0.350 | 0.851 | <0.001 |
|  | Hand Opening & Closing | round 1 | 0.378 | 0.193 | 0.608 | <0.001 |
|  |  | round 2 | 0.48 | 0.223 | 0.784 | <0.001 |
|  | Wrist Pronation Supination | round 1 | 0.275 | 0.117 | 0.504 | <0.001 |
|  |  | round 2 | 0.822 | 0.645 | 0.943 | <0.001 |
| **Left** | Resting Tremor | round 1 | 0.647 | 0.473 | 0.81 | <0.001 |
|  |  | round 2 | 0.422 | 0.168 | 0.749 | <0.001 |
|  | Postural Tremor | round 1 | 0.127 | 0.003 | 0.34 | <0.001 |
|  |  | round 2 | 0.41 | 0.16 | 0.41 | <0.001 |
|  | Kinetic Tremor | round 1 | 0.434 | 0.24 | 0.658 | <0.001 |
|  |  | round 2 | 0.255 | 0.063 | 0.596 | <0.001 |
|  | Finger Tapping | round 1 | 0.231 | 0.086 | 0.454 | <0.001 |
|  |  | round 2 | 0.468 | 0.213 | 0.774 | <0.001 |
|  | Hand Opening & Closing | round 1 | 0.275 | 0.117 | 0.504 | <0.001 |
|  |  | round 2 | 0.822 | 0.645 | 0.943 | <0.001 |
|  | Wrist Pronation Supination | round 1 | 0.458 | 0.271 | 0.675 | <0.001 |
|  |  | round 2 | 0.445 | 0.197 | 0.759 | <0.001 |

**Table 6:** ICCs between raters for round 1 and round 2 for each movement, for the right hand and the left hand (considering only the same six raters in both rounds).
